# Sub-cellular Electrical Heterogeneity Revealed by Loose Patch Recording Reflects Differential Localization of Sarcolemmal Ion Channels in Intact Rat Hearts

**DOI:** 10.3389/fphys.2018.00061

**Published:** 2018-02-13

**Authors:** Igor V. Kubasov, Andrei Stepanov, Danila Bobkov, Przemysław B. Radwanski, Maxim A. Terpilowski, Maxim Dobretsov, Sandor Gyorke

**Affiliations:** ^1^I. M. Sechenov Institute of Evolutionary Physiology and Biochemistry RAS, Saint-Petersburg, Russia; ^2^Institute of Cytology RAS, Saint-Petersburg, Russia; ^3^Dorothy M. Davis Heart and Lung Research Institute, College of Medicine, Ohio State University, Columbus, OH, United States; ^4^Division of Pharmacy Practice and Science, College of Pharmacy, Ohio State University, Columbus, OH, United States; ^5^Department of Anesthesiology, University of Arkansas for Medical Sciences, Little Rock, AR, United States

**Keywords:** T-tubule, excitation-contraction coupling, action potential, ion channels and transporters, cardiac muscle

## Abstract

The cardiac action potential (AP) is commonly recoded as an integral signal from isolated myocytes or ensembles of myocytes (with intracellular microelectrodes and extracellular macroelectrodes, respectively). These signals, however, do not provide a direct measure of activity of ion channels and transporters located in two major compartments of a cardiac myocyte: surface sarcolemma and the T-tubule system, which differentially contribute to impulse propagation and excitation-contraction (EC) coupling. In the present study we investigated electrical properties of myocytes within perfused intact rat heart employing loose patch recording with narrow-tip (2 μm diameter) extracellular electrodes. Using this approach, we demonstrated two distinct types of electric signals with distinct waveforms (single peak and multi-peak AP; AP1 and AP2, respectively) during intrinsic pacemaker activity. These two types of waveforms depend on the position of the electrode tip on the myocyte surface. Such heterogeneity of electrical signals was lost when electrodes of larger pipette diameter were used (5 or 10 μm), which indicates that the electric signal was assessed from a region of <5 μm. Importantly, both pharmacological and mathematical simulation based on transverse (T)-tubular distribution suggested that while the AP1 and the initial peak of AP2 are predominantly attributable to the fast, inward Na^+^ current in myocyte's surface sarcolemma, the late components of AP2 are likely representative of currents associated with L-type Ca^2+^ channel and Na^+^/Ca^2+^ exchanger (NCX) currents which are predominantly located in T-tubules. Thus, loose patch recording with narrow-tip pipette provides a valuable tool for studying cardiac electric activity on the subcellular level in the intact heart.

## Introduction

In the heart, the action potential (AP) generated in the sinoatrial node propagates trough myocardial syncytium to induce coordinated contraction of myocytes composing the heart. There are two major compartments in cardiac myocyte membrane: the surface sarcolemma and T-tubules. These two compartments are populated by different sets of ion channels and transporters that contribute differently to AP propagation and myocyte contraction (Franzini-Armstrong et al., 1998; Brette and Orchard, 2007; Scriven et al., 2013). Thus, the fast Na^+^ channels (Na_v_ 1.5) responsible for AP propagation appear to be localized mainly on the surface sarcolemma (Maier et al., 2002; Brette and Orchard, 2006; Lin et al., 2011; Bhargava et al., 2013; Westenbroek et al., 2013), while the L-type Ca^2+^ channels (LTCC) and Na^+^/Ca^2+^ exchange (NCX) involved in cardiac excitation contraction coupling (Niggli and Lipp, 1995; Poláková et al., 2008; Ottolia et al., 2013; Eisner et al., 2017) were reported to reside preferentially in the membrane of the T-tubules (Frank et al., 1992; Kieval et al., 1992; Leech and Holz, 1994; Sun et al., 1995; Scriven et al., 2000; Balycheva et al., 2015). To further add to this complexity, the relative prominence of ion transport systems, such as NCX, in surface vs. T-tubule sarcolemma may vary between different species (Gadeberg et al., 2017), different anatomical and histological areas of the heart (Gómez et al., 1997) and cardiac pathologies along with the stage of disease progression (Balijepalli et al., 2003; Banyasz et al., 2008; Dibb et al., 2009; Zima et al., 2014; Radwanski et al., 2016; Veeraraghavan et al., 2017; Yue et al., 2017). Therefore, developing a better appreciation for the contribution of ionic currents from the outer membrane and T-tubular regions to cardiac AP is a critical for understanding normal heart function as well as under pathologic conditions.

Whole cell patch clamp or intracellular microelectrode recording in isolated cell or multicellular preparations (Knisley and Neuman, 2003; Mačianskiene et al., 2015) provide information on integral activity of ion channels and transporters of the plasma membrane of entire myocyte only. These signals lack information about the specific contribution of sarcolemmal and T-tubular compartments to the recorded signal. While the inside-out patch mode permits registration of localized channel activity (Leech and Holz, 1994), the channels located in the excised membrane patch are studied outside of their native environment. This method is limited to data acquisition from the surface sarcolemma, and cannot be used for electrophysiological studies on the T-tubular membrane.

Extracellular recording from the whole heart using metal or electrolyte solution-filled glass pipette electrodes (“loose patch”) offers a useful approach for studying the properties of the cardiac action potential and its underlying ion mechanisms under conditions when the functional and structural integrity of the myocardium is preserved (Franz, 1983; Franz et al., 1984; Knollmann et al., 2002). In particular this method permits recording from anatomically specific areas from the myocardial surface during propagating AP propagation. Most of such studies, however, have been performed with extracellular macroelectrodes with the tip diameter of 100–150 μm (Eickhorn et al., 1994; Ramos-Franco et al., 2016) permitting investigation of integral responses of thousands of myocytes but not individual cells, let alone subcellular compartments. Thus, information on domain-specific electrical activity and its underlying ionic mechanisms in cardiac myocytes is lacking.

Recently, using loose patch extracellular recording with small diameter (2–5 μm) microelectrodes we demonstrated that skeletal muscle fibers exhibit two types of propagating APs. The observed differences were suggested to reflect heterogeneous distribution of certain ion transport systems, including K^+^ channels, in the membrane of the T-tubule system and sarcolemma (Kubasov and Dobretsov, 2012). As stated above, similar to skeletal muscle fibers, a well-developed T-tubular system is present in cardiac myocytes to support cardiac EC coupling (Franzini-Armstrong et al., 1998; Soeller and Cannell, 1999). However, the electrical properties of this membrane system and functional activities of the resident ion transport systems remain to be defined. Such information would be especially valuable if obtained in settings of physiologically relevant AP activation in intact myocardium.

Therefore, the goal of the present study was to investigate the applicability of the loose patch method for extracellular recording of propagating APs in ventricular myocytes on the surface of isolated rat hearts. This approach was used to determine spatiotemporal properties of AP propagation and the underlying ionic mechanisms for the recorded responses. Our study showed that the AP exhibit clear spatial heterogeneity attributable to activity of several key ion transport mechanisms of the sarcolemma, including Na^+^ channels, Ca^2+^ channels, and Na^+^/Ca^2+^ exchange, distributed differently between the surface and T-tubule membranes of the cardiac myocyte. Our study showed that the AP exhibit clear spatial heterogeneity at least in part attributable to non-uniform distribution of voltage-dependent Na^+^ and Ca^2+^ channels and NCX transporters between the surface and T-tubule membranes of the cardiac myocyte.

## Materials and methods

Studies were conducted in accordance with regulations of the National Committee on Bioethics of the Russian Academy of Sciences. The protocol was approved by the Ethics Committee of the Sechenov Institute of Physiology and Evolutionary Biochemistry. Experiments were performed in hearts isolated from adult Wistar male rats (*N* = 28; body weight 200–250 g). Rats were anesthetized with sodium pentobarbital (80 mg kg^−1^, i.p.). Hearts were harvested via thoracotomy and retrogradely perfused through the aorta with a Tyrode's solution containing (in mM): 140 NaCl, 1.0 MgCl_2_, 4.5 KCl, 10 glucose, 1 CaCl_2_ and 10 HEPES, at pH 7.3, gassed with 95% O_2_ and 5% CO_2_ (at temperature of 36–37°C). To improve access of electrodes to the external layer of cardiomyocytes, hearts were treated with collagenase. Treatment with collagenase substantially improved the quality of traces (amplitude, signal to noise ratio) without altering the overall shape and time course of the responses. For collagenase treatment, Langendorff-perfused hearts were immersed in a Tyrode's solution complemented with collagenase (0.8 mg/ml, Sigma, USA) for 10–15 min (at temperature of 36–37°C). After collagenase treatment hearts were placed in a chamber for electrophysiological recording. Myosin ATPase inhibitor, blebbistatin (10 μM, Sigma, USA) was added to the Tyrode's solution perfusing the heart to stop heart contraction and prevent associated artifacts (Farman et al., 2008).

**Figure 1:**
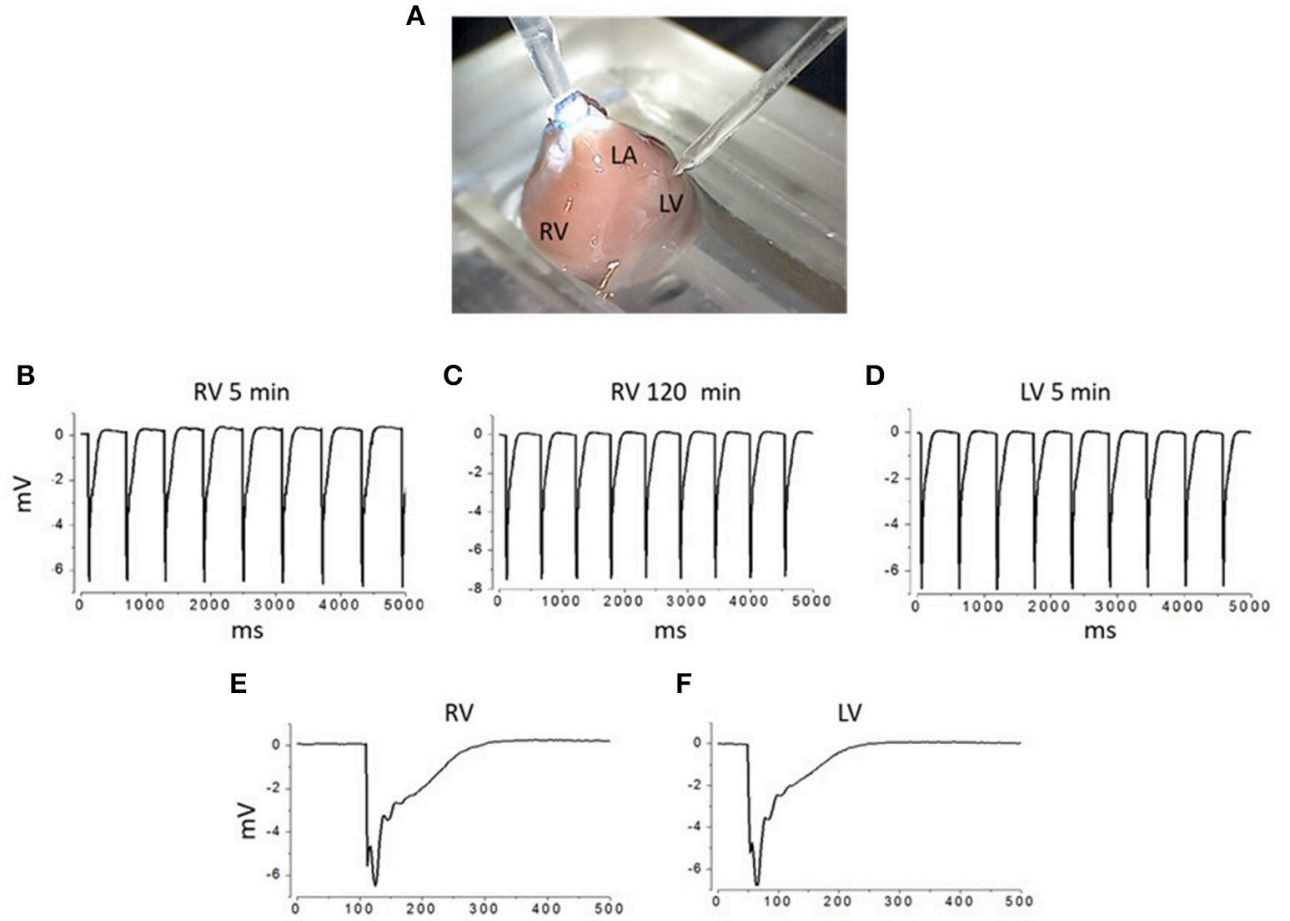
Experimental chamber and examples of extracellular recordings from different areas of interest on the surface of isolated rat heart APs. **(A)** Image of an isolated heart in the experimental chamber. Right and left ventricle (RV and LV) and left atrium (LA) are labeled. Recording electrode is approaching LV from the top right corner of the image. Reference electrode (silver plate) is positioned at the bottom of the chamber, filled with Ringer solution. **(B–D)** Examples of extracellular recordings of spontaneous APs from the right ventricle (obtained at 5th and in the same site at 120th min after the initial contact between electrode and the heart surface was established; **B,C**, respectively) and from the left ventricle of the same heart. **(E,F)** Examples of individual APs recorded from right and left ventricle **(E,F)**. All recordings were done using 5 μm outer tip diameter pipettes.

Microelectrodes with a tip outer diameter (OD) of 2, 5, and 10 μm were pulled using a programmable puller (Sutter Instruments, Model P-1000, USA) and fire-polished. The electrodes were filled with Tyrode's solution and had resistance in the range 4.2–5.0, 2.1–2.5, and 1.1–1.5 MΩ, for 2, 5, and 10 μm pipettes, respectively. The pipette tip was brought into contact with the surface of the heart within the area of interest using a mechanical manipulator NMH-21 (Narishige, Japan; Figure 1A). Seal resistance after formation of the contact, i.e., loose patch (no suction applied), ranged from 30 to 55 MΩ (2 μm pipettes), 15 to 25 MΩ (5 μm pipettes), and 3 to 6 MΩ (10 μm pipettes). The extracellular electric signal was recorded during SA node-driven rhythmic activity. As a rule, recordings were performed from 10 to 20 sites of the myocardial surface in course of a sequential repositioning of the electrode from one site to another within an area of 1 × 1 mm. Recorded signals were amplified, filtered (at 0.03–10 kHz) by AM-1500 amplifier (A-M Systems, USA) and digitized at 10 μs intervals using a 16-bit analog–digital converter NI USB-6211 (National Instruments, USA). Recorded signals were stored on a computer hard-drive and then analyzed off-line with Clampfit 6.0 (Axon Instruments, USA). The waveform amplitudes were measured from baseline to the peak. We used the standard method of extracellular recording with narrow-tip pipettes, referred as “focal current recording”, previously used for mapping spatial distribution of electrical signals along nerve terminals (Brigant and Mallart, 1982; Wolters et al., 1994) and muscle fibers (Stuhmer et al., 1983; Kubasov and Dobretsov, 2012). The signals recorded with this method represent electrical potential changes across the seal resistance, which is directly proportional to the local currents generated predominantly under the tip of the pipette (Mallart, 1985; Wolters et al., 1994; Bennett et al., 2000; Kubasov and Dobretsov, 2012). Examples of recordings of rhythmic waveforms obtained from the right and left ventricle recorded with a 5 μm tip OD pipette are illustrated in Figures 1B–F. Most of the data were obtained from the surface of the middle portion of the right ventricle. The recordings from any particular heart location were stable, having their frequency and waveform shape and amplitude preserved for at least 1 h of experiment (Figures 1B,C). In studies with inhibitors of Na^+^ and Ca^2+^ channels or NCX, the corresponding blockers, tetrodotoxin (TTX, Sigma, USA), nifedipine (Sigma, USA), or benzyloxyphenyl derivative SN-6 (Tocris, USA), were added to the recording pipette's solution. For imaging T-tubule structure, hearts were stained with Di-8-ANEPPS, (Molecular Probes, USA) delivered through perfusion with a Tyrode's solution containing 30 μM of the dye for 30 min at 36–37°C.

The membrane structure of epicardial myocytes was analyzed *in situ* by confocal microscopy [Leica SP5 equipped with × 63 (NA = 1.4) oil-immersion lens]. The epicardial images were used to define the distribution of T-tubule openings within 60 randomly selected standard in size (90 × 90 pixels) regions of interest (ROI) on the myocyte surface. To map the location of T-tubular openings, each of these ROIs was processed using a Find Maxima protocol (open-source ImageJ software; https://imagej.nih.gov/ij/download.html) with noise tolerance level set to 3. T-tubule opening regions were selected by their excessive deviation from the noise level. The location of each T-tubule opening on a ROI was marked by a dot (single pixel) corresponding to the location of the center of mass of the respective T-tubule opening area. Generated in this fashion ROI templates were then used for Monte Carlo simulation to estimate the probability of pipettes of different tip internal diameter randomly positioned on myocyte surface to cover membrane patches containing one or more T-tubule openings (P_t_). The simulation protocol consisted of 50 × 10^6^ iterations per template per tip diameter in which the position of the electrode tip (i.e., circle with a given ID) over the template was randomly varied. The fraction of iterations in which one or more T-tubule openings were found to within, or at the border, of the circle was counted and used for calculation of P_t_ for a given pipette ID. This procedure was repeated for all 60 templates (ROIs) and final P_t_ value was calculated as average of individual template P_t_ values. The protocol was written using Python 3.6 Numpy (python.org) programming language. Finally, confocal microscopy in combination with the fluorescent dye fluorescein (to label the interior of the pipette) was used to define the internal diameter (ID) of the 2 μm recording pipettes.

### Statistical analysis

All data were tested for normality of distribution (Shapiro-Wilk test) and, based on the results of this test, analyzed using non-parametric repeated measures Friedman tests followed by Dunn's multiple comparisons testing. *P* < 0.05 was considered to indicate a statistically significant difference. Statistical analysis was conducted using Origin 5.0 (OriginLab Corporation, Northampton, MA, USA) and Prizm 5.0. (GraphPad Software, Inc., La Jolla, CA, USA) graph and statistical software. Values are expressed as the mean ± standard error (SE).

## Results

**Figure 2:**
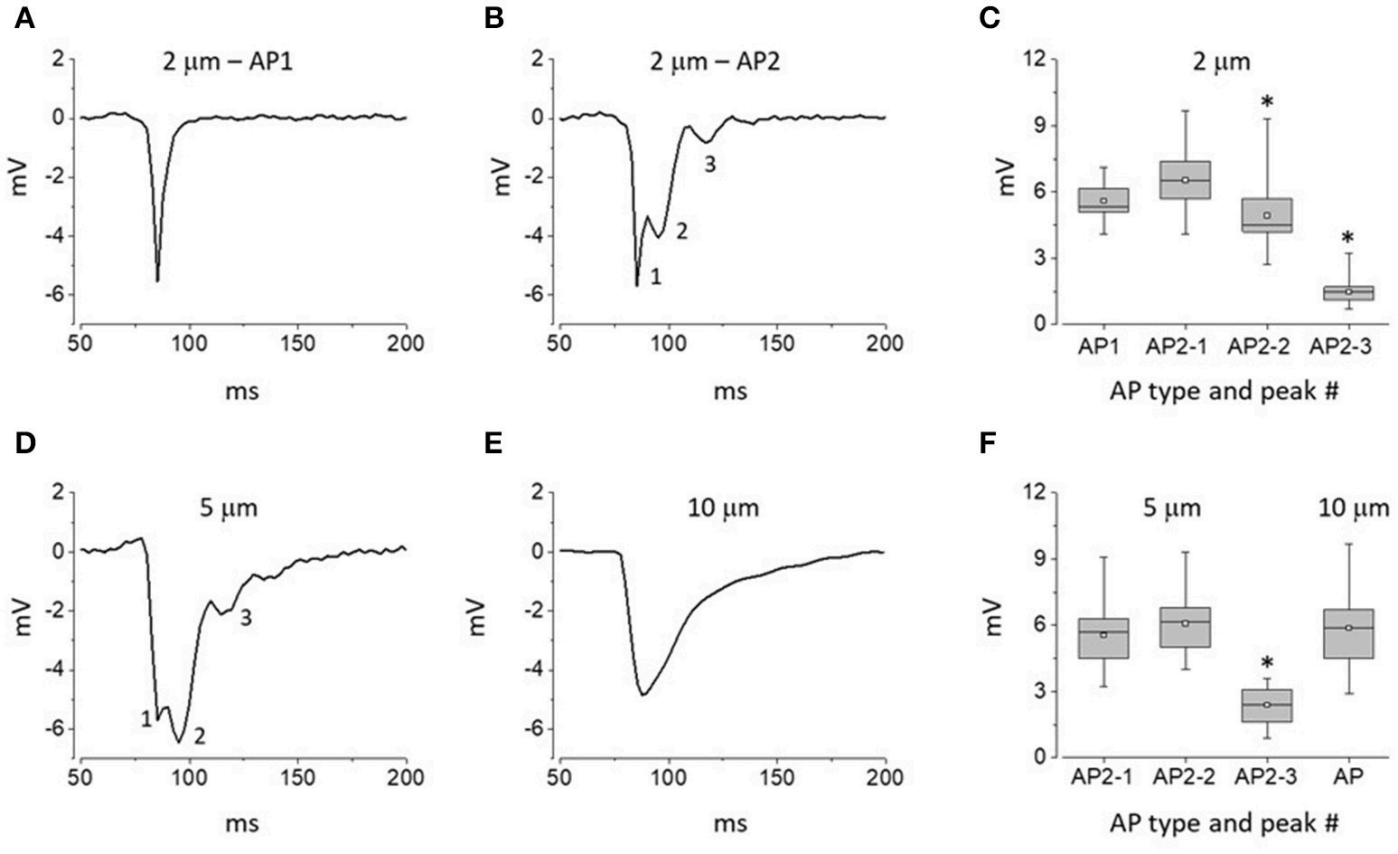
Distinct waveforms of APs recorded at the surface of intact hearts with electrodes of different tip diameter. **(A,B)** Representative traces of APs recorded with 2 μm pipettes: AP1 and AP2 (**A,B**, respectively). **(C)** Whisker box plots of peak amplitudes of AP1 and AP2 waveforms recorded with 2 μm pipettes. Data represent averages ± SE for 40 AP1 and 190 AP2 sites (8 hearts). **(D,E)** Representative traces of APs recorded with 5 μm **(D)** and 10 μm pipettes **(E). (F)** Whisker box plots of peak amplitudes of response waveforms recorded with 5 and 10 μm pipettes (146 and 95 recordings of APs using 5 and 10 μm tip diameter pipettes, respectively; 8 hearts per group). At least 3 distinct peaks could be distinguished within type 2 (AP2) response waveforms; see numerical labels next to traces shown in **(B,C)**. Asterisks in **(A,C)** indicate statistically significant difference between amplitudes of the first and given AP2 components (Repeated measures Friedman test followed by Dunn's test for multiple comparisons; *p* < 0.01). In whisker box plots the 25 and 75% values are represented by lower and upper box borders, respectively; median and mean values are represented by horizontal bars and squares within the box, and whisker length is determined by minimal and maximal data values within the corresponding datasets.

We used extracellular recording with narrow-tip electrodes to assess spatial heterogeneities in electrical activity at the myocardial surface in Langendorff-perfused rat hearts. An electrode with a tip diameter of 2 μm was repeatedly brought to the surface of the ventricle to record series of propagating APs at spatially distinct but adjacent sites. The signals recorded with this technique were substantially different from the classical cardiac intracellular APs, monophasic APs recorded previously with extracellular macroelectrodes or optical APs (Knollmann et al., 2002; Knisley and Neuman, 2003; Radwanski et al., 2010; Mačianskiene et al., 2015; Ramos-Franco et al., 2016). The recorded APs exhibited a significant variability in waveform from one recording site to another. In general, two types of waveforms were recorded: relatively rare (17%) single peak APs and more commonly (83%) complex, multi-peak APs. In this study these two distinct waveforms are designated as APs of types 1 and 2, respectively (Figures 2A,B; total 230 recordings, 8 hearts). The single peak AP1 signals represented fast signals with a short rise time and a fast decay (Figure 2A). Complex AP2 signals were characterized by presence of at least three distinct peaks (Figure 2B). While the amplitude of the initial component of AP2 was similar to those of the single-peak AP1 signal, the later components exhibited progressively smaller amplitude compared to the initial peaks (Figure 2C).

To further probe heterogeneity of extracellular signals, we performed experiments with larger tip diameter electrodes of 5 and 10 μm. In experiments with 5 μm tip diameter electrodes we did not observe AP1 waveforms. Notably, all recorded signals were multiphasic signals resembling type 2 APs described above (Figure 2D; 146 recordings, 8 hearts). However, while preserving the overall multi-peak waveform, the APs recorded with 5 μm tip diameter pipettes showed a significant augmentation of the late components compared to those recorded with 5 μm tip pipettes (Figures 2C,F). In experiments with 10 μm-tip diameter electrodes all recorded signals (95 recordings, 8 hearts) were represented by a slow monophasic integral signal (Figure 2E). Taken together these results suggest that the myocardium exhibits microscopic heterogeneity in electrical activity on a spatial scale of <5 μm).

Extracellular recordings represent electrical events occurring mainly under and in the immediate vicinity of the tip of the recording pipette (Mallart, 1985; Wolters et al., 1994; Bennett et al., 2000; Kubasov and Dobretsov, 2012). Therefore, one of the reasons for the heterogeneity of signals described above could be the position of the electrode relative to opening of T-tubule(s) and the rest of the sarcolemma where different pools of ion channels and transporters reside. For example, it is well established that the fast Na^+^ channels (Na_v_1.5) are distributed mainly throughout the surface of sarcolemma, while other key ion transport systems that include the L-type Ca^2+^ channels and NCX are preferentially localized to the T-tubules (Sun et al., 1995; Scriven et al., 2000; Radwanski et al., 2016). Therefore, using 5 μm-tip electrodes and selective pharmacological inhibitors applied locally to the recording site via pipette solution, we examined the contribution of various ionic transports to the reported AP heterogeneity.

**Figure 3:**
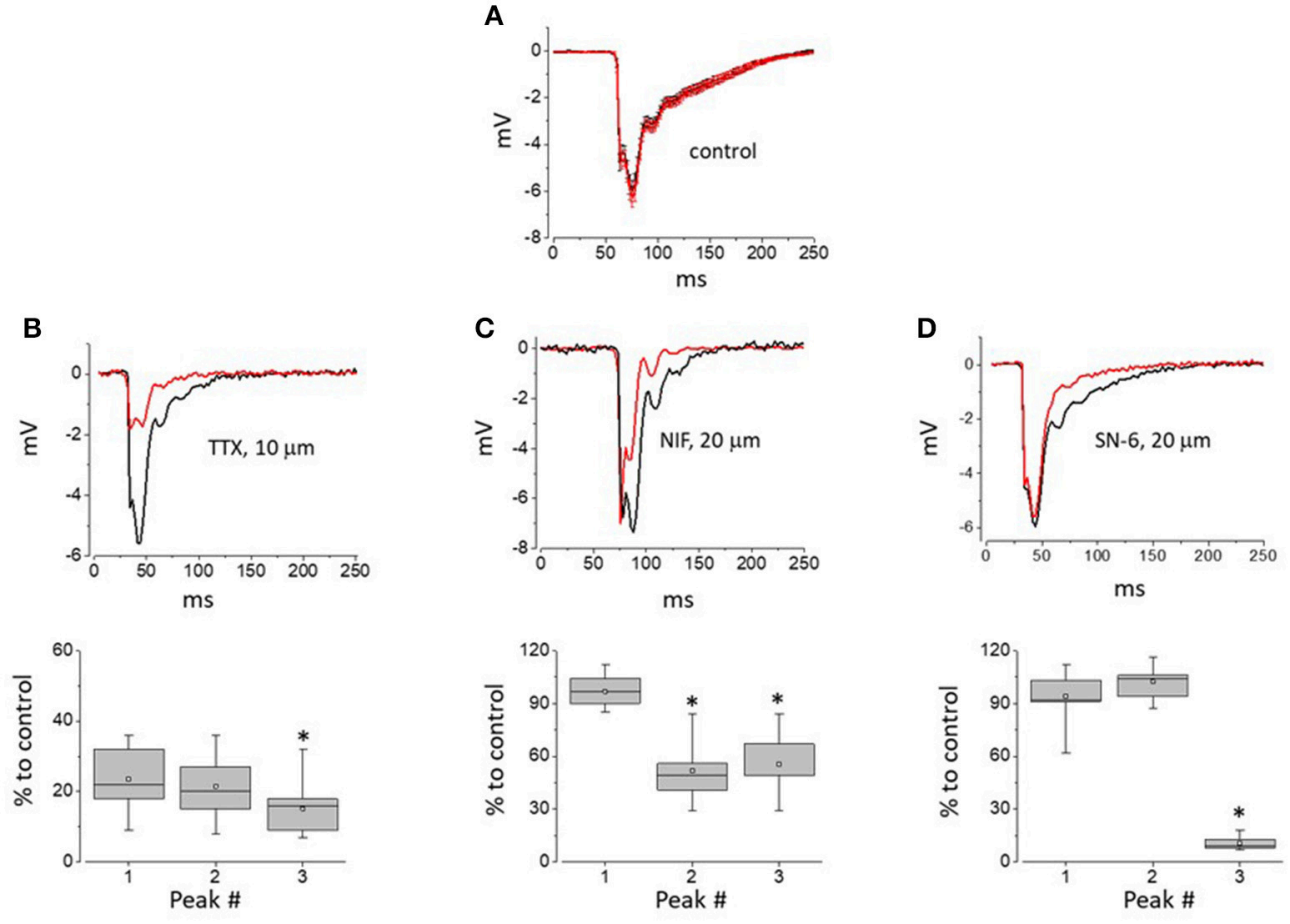
The impact of Na^+^ and L-type Ca^2+^ channel as well as NCX inhibition on the components of APs recorded with 5 μm-thick pipettes. **(A)** AP2 recorded within 5 min after initial myocyte-pipette contact was established and during 25–30 min of experiment (black and red traces, respectively). Traces shown are point-by-point averages (±SE) of control recordings obtained from 12 independent sites (4 hearts) and synchronized by the descending phase of their first negative peak. **(B–D)** Top row—Representative traces of AP obtained within the first 5 min and following 25–30 min of continuous recording with pipettes loaded with solutions containing 10 μM TTX, 20 μM Nifedipine, or 20 μM SN-6, prospectively. Bottom row—whisker box plots (52, 38, and 45 recordings for TTX, Nifedipine, and SN-6, respectively; 4 hearts per group) of individual peaks of AP2 measured during 25–30 min of recording performed with pipettes loaded with solutions containing TTX **(B)**, Nifedipine or SN-6 **(D)**, and expressed as percentage of amplitudes of respective peaks measured within first 5 min of recording. Asterisks indicate statistically significant difference between relative amplitudes of the first and given AP2 components (Repeated measures Friedman test followed by Dunn's test for multiple comparisons; *p* < 0.01). In whisker box plots the 25 and 75% values are represented by lower and upper box borders, respectively; median and mean values are represented by horizontal bars and squares within the box, and whisker length is determined by minimal and maximal data values within corresponding dataset.

As demonstrated in Figure 3A, AP waveform in control experiments using 5 μm pipettes filled with physiological saline was stable over time during long-term recordings. However, addition of TTX to the intra-pipette solution resulted in progressive suppression of all the peaks composing recorded with such pipettes AP2s (Figure 3B). Unlike that, local application of the LTCC blocker, nifedipine resulted in the inhibition of the recorded signal second and third peaks only (Figure 3C). Finally, local application of SN-6, an NCX inhibitor, had relatively minor effect on the first and second peaks of the AP2, but resulted in a marked suppression of its later components (Figure 3D). Taken together these results suggest that while the initial peak of the multicomponent extracellular APs is a result of fast Na^+^ channel activity, the second and later peaks correspond, at least in part, to LTCC and NCX activity, respectively.

**Figure 4:**
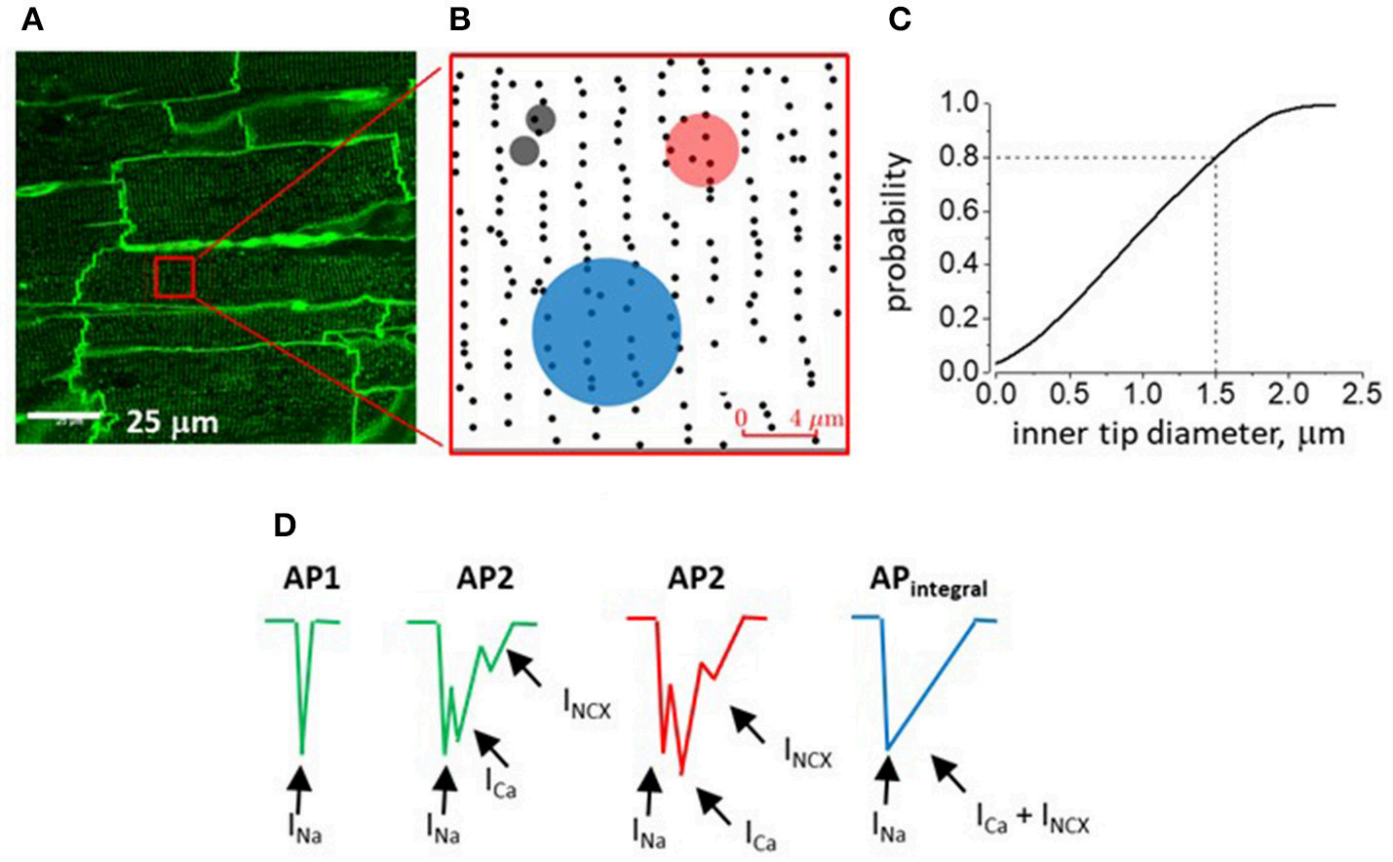
Confocal imaging of myocardium of intact rat heart and analysis of T-tubular opening density. **(A)** Representative image from the surface of perfused intact rat heart stained with Di-8-ANEPS to outline T-tubules (representative of 24 images from the middle part of the surface of RV; Figure 1A). **(B)** Region of interest (ROI; see red square in **A**) after processing for detection T-tubule openings density (black dots). Semitransparent black, red and blue circles represent experimentally measured areas covered by the inner rim of pipettes of 2, 5, and 10 μm-thick pipettes (ID values are 1.5 ± 0.2, 3.9 ± 0.2, and 7.9 ± 0.3 μm, respectively). **(C)** Monte Carlo simulation of the probability of a randomly positioned electrode with given inner tip diameter to cover a myocyte region containing at least one t-tubular opening. During simulation, the pipette tip inner diameter was increased starting from 0.05 to 2.5 μm in steps of 0.05 μm (50 steps) and the probability of interest was calculated based on the results of 10^6^ protocol iterations per each step. **(D)** Schematic presentation of components of AP waveforms recorded with 2 μm (green; T-tubule-free and T-tubule-containing myocyte region; AP1 and AP2, respectively), 5 and 10 μm outer tip diameter electrodes (red, AP2 and blue, AP_integral_ drawings, respectively). Hypothetically, different currents contribute differentially to formation of individual peaks/components of recorded signals (arrows with respective labels).

To further assess the possibility that the observed AP heterogeneities reflect the activity of ion transport mechanisms specifically residing in the T-tubules we set out to estimate the probability of the pipette tip lumen covering a membrane patch containing at least one T-tubule opening (P_t_). T-tubule distribution was defined by confocal imaging with the membrane dye Di-8-ANEPPS under experimental conditions similar to those used in electrophysiological studies (Figure 4A). Randomly selected regions of interest (ROI; see Figures 4A,B and section Materials and Methods) were used as templates for Monte Carlo simulation of probability (P_t_) of the pipette tip of certain internal diameter (ID) randomly hitting at least one T-tubule opening on myocyte surface. Based on the obtained P_t_ vs. pipette ID relationship curve (Figure 4C), P_t_ is 50% with a pipette ID of 1 μm, and P_t_ is 100% when pipettes with ID ≥ 2.2 μm are used. To measure the internal tip diameter of our recording pipettes the pipettes were filled with a solution containing fluorescein and their tip opening was imaged with a confocal microscope. For our 2 μm OD pipettes these measurements yielded an average ID of 1.5 ± 0.2 μm, which corresponds to P_t_ = 0.8 (Figure 4C, dashed lines). Notably, this estimate value is close to the probability of recording AP2 with same internal diameter electrodes (0.83). These results support the possibility that AP2 reflects electrical activity of the T-tubule membrane.

## Discussion

In the present study we investigated electrical properties of myocytes within intact rat hearts employing loose patch recording with narrow-tip diameter (2–5 μm) extracellular electrodes. Using this approach, we demonstrated for the first time that propagating APs in cardiac myocytes exhibit significant subcellular heterogeneity as evidenced by presence of two distinct types of signals: single-peak (AP1) and multi-peak (AP2), respectively. Importantly, this heterogeneity of signals was lost upon increasing pipette tip diameter from 2 to 5 or 10 μm, indicating AP variations on a spatial scale of <5 μm. Our results are consistent with the possibility that this heterogeneity in electrical activity results from differential localization of Na^+^ channels, LTCC, and NCX between the surface sarcolemma and T-tubules.

Using pharmacological analysis, we gained insights into the ionic basis of the components of AP1 and AP2. These experiments suggested that AP1 and the initial fast component of AP2 can be attributed to TTX-sensitive Na^+^ current carried by the cardiac-type Na_v_1.5 present mainly on the surface of sarcolemma (Maier et al., 2002, 2004; Brette and Orchard, 2006; Bhargava et al., 2013; Westenbroek et al., 2013; Radwanski et al., 2015). At the same time, based on the inhibitory effects of nifedipine and SN-6, the second and subsequent components of AP2 likely reflect currents carried by the LTCC and NCX. These results are consistent with previous studies which demonstrated that LTCC and NCX predominantly reside in T-tubules (Frank et al., 1992; Sun et al., 1995; Scriven et al., 2000; Radwanski et al., 2016).

The differential contribution of ion currents present in T-tubules to the AP1 vs. AP2 waveforms is also consistent with the results of analysis of the effect of pipette geometry on the probability of the pipette covering a membrane region containing T-tubule opening(s). Assuming that the signal recorded by 2 μm-thick (1.5 μm ID) electrodes represents mainly the activity of electrogenic mechanisms located within the membrane patch under the rim of the electrode, the probability of recording from one or more T-tubules estimated based on results of Monte Carlo simulation closely matches that expected from the relative frequency of type 2AP waveforms observed in corresponding experiments (~80%; see Figure 4C; dashed lines). It may also be inferred from simulation studies (Figure 4C) that electrodes with internal tip diameter larger than 2.2 μm will always cover at least several T-tubule openings, thus accounting for AP2 multiphasic waveform. Unlike AP2 recorded with 2 and 5 μm-thick pipettes (ID is 1.5 and 3.9 μm, respectively), the waveforms recorded with 10 μm pipettes (ID is 7.6 μm) present smooth, single peak signals. This could be attributed to a combination of several factors, including asynchronous activation of channels residing in multiple T-tubules (50–60) as well as the lower seal resistance and greater capacitance of large tip diameter pipettes resulting in effective shunting and smoothing individual peaks. Accordingly, slow and smooth AP waveforms were recorded with larger-diameter tip (250 μm) pipettes by others (Knollmann et al., 2001; Ramos-Franco et al., 2016).

The current findings and their interpretation are in general agreement with the results of our previous studies conducted in skeletal muscle. Using a similar approach we recently demonstrated two types of APs potentially attributable to differential distribution of ion channels and transporters on the surface sarcolemma and T-tubule system of muscle fibers (Kubasov and Dobretsov, 2012). However, the waveforms of extracellular AP recorded in skeletal and cardiac muscles vary substantially. While the signals in skeletal muscle were characterized by the presence of either two or three phases (“positive-negative,” AP1 and “positive- negative-positive,” AP2, respectively), the cardiac APs were presented by single-peak (AP1) or multi-peak (AP2) responses, all negative, waveforms. Tentatively, some of these differences could be ascribed to different composition of ion transport systems in the sarcolemma, including the T-tubules, of skeletal muscle fibers and cardiac myocytes. For example, the late component of AP2 recorded in skeletal muscle appears to be dominated by outward current through T-tubular K^+^ channels (Wolters et al., 1994; Kubasov and Dobretsov, 2012). The same component of the cardiac AP2 is likely to be dominated by inward currents via LTCC and NCX, known to be preferentially expressed in cardiac myocyte T-system (Frank et al., 1992; Sun et al., 1995; Scriven et al., 2000; Ferreiro et al., 2012). The difference in skeletal muscle and cardiac APs in the magnitude of their first, positive, capacitive phase is likely to be explained by differences in the recording techniques. In skeletal muscle experiments (Kubasov and Dobretsov, 2012) the electrode was immersed into the bath solution to the depth of 1–2 mm and therefore its tip capacitance is expected to be higher and taking longer to charge as compared to the electrode pressed against the wet surface of Langendorff-perfused heart in the current study.

While differential expression of Na^+^ and Ca^2+^ channels and NCX between the myocyte surface sarcolemma and T-tubular membrane appears to be the simplest explanation for the observed heterogeneity of extracellularly recorded cardiac AP waveforms (Figure 4D), our study has certain limitations. In addition to NCX assessed in this study, activity of other ion channels and transporters such as various K^+^ channels and Na^+^/K^+^-ATPase is likely to contribute to the late stages of recorded AP waveforms and needs to be studied. Furthermore, Na^+^ channels of neuronal type (TTX-sensitive) have been reported to be abundantly expressed in the T-tubular membrane (Györke et al., 2017; Veeraraghavan et al., 2017) and thus the activity of these channels may also add to the generation of the AP2 waveform. Finally, as another potential concern, the commonly used pharmacological reagent SN-6 was employed to inhibit NCX. SN-6 has been shown to affect targets other than NCX (Niu et al., 2007; Gandhi et al., 2013); therefore, we cannot rule out the possibility that this reagent exerts off-target effects in our experiments.

In summary, the main finding of this study is that extracellular recording from Langendorff-perfused hearts with narrow-tip pipettes can be applied to study myocyte electrical activity at the subcellular level under physiological settings. Measurement of the distinct components of the single and multi-peak extracellular waveforms recorded with 2–5 μm pipettes may provide a reliable and simple approach for elucidation of local ion transport and signaling mechanisms residing in the surface sarcolemma and T-tubule compartment of various myocardial regions in both atria and ventricles. This approach may also be useful for gaining insights into ionic and structural alterations of T-tubules and other discrete subcellular domains in cardiac disease. Although this study was carried out on rat hearts, the presented approach should be also applicable to Langendorff-perfused hearts of other species.

## Author contributions

IK, PR, and SG designed the study. IK, DB, and AS collected the electrophysiological and imaging data. IK and MD analyzed the electrophysilogical and optical imaging data. MT and MD analyzed the optical imaging data. MT performed mathematical modeling. IK, PR, MD, and SG interpreted the data. IK and SG wrote the manuscript. MD and PR edited the manuscript.

### Conflict of interest statement

The authors declare that the research was conducted in the absence of any commercial or financial relationships that could be construed as a potential conflict of interest.
